# Primary Testicular Extranodal NK/T-cell Lymphoma Nasal Type Associated With Epstein-Barr Virus Infection: A Moroccan Case Report

**DOI:** 10.7759/cureus.63361

**Published:** 2024-06-28

**Authors:** Hanaa Nejjari, Intissar Ait Zine, Wafa Ammouri, Zakia Bernousssi, Hakima Kabbaj

**Affiliations:** 1 Central Laboratory of Virology, Ibn Sina Hospital, Faculty of Medicine and Pharmacy, Mohammed V University, Rabat, MAR; 2 Internal Medecine Department, Ibn Sina Hospital, Faculty of Medicine and Pharmacy, Mohammed V University, Rabat, MAR; 3 Pathology Department, Ibn Sina Hospital, Faculty of Medicine and Pharmacy, Mohammed V University, Rabat, MAR

**Keywords:** real-time pcr, testis, extranodal nk-t-cell, lymphoma, epstein-barr virus

## Abstract

Epstein-Barr virus (EBV) is a ubiquitous and potentially oncogenic human herpesvirus. In this article, we discuss a rare case of primary testicular NK/T-cell lymphoma, nasal type, associated with high plasma Epstein-Barr virus DNA load. The patient, a 45-year-old man, presented with painful testicular swelling, fever, and weight loss. An orchiectomy revealed tumor proliferation, which was diagnosed as testicular NK/T cell lymphoma, nasal type, confirmed by immunohistochemistry, and classified as stage IV according to the Ann Arbor classification. The patient was treated with SMILE chemotherapy. After treatment, a PET scan showed complete remission with negative EBV DNA levels. The discussion focused on the role of EBV in the development of this malignant lymphoproliferation and the importance of quantifying EBV DNA load by real-time PCR in assessing prognosis, patient follow-up, and response to treatment.

## Introduction

Epstein-Barr virus (EBV) is a ubiquitous and potentially oncogenic human herpes virus that was first isolated from a cultured B-cell lymphoma in 1964 [1-3]. It may be involved in the development of some malignant lymphoproliferative disorders that predominantly affect B cells, including Burkitt's lymphoma, Hodgkin's lymphoma, and diffuse large B-cell lymphoma. Some lymphomas originate from the persistence of the EBV in the B lymphoid system. The virus remains latent in B cells and reactivates under certain conditions, leading to uncontrolled cell growth. In other cases, lymphomas arise when EBV infects unnatural target cells, leading to abnormal proliferation and tumor formation [4]. EBV can infect T cells and natural killer (NK) cells, leading to lymphoproliferative diseases such as systemic T-cell-positive childhood lymphoma, aggressive NK cell leukemia, EBV-positive primary T/NK cell lymphoma, and extranodal nasal-type NK/T cell lymphoma (ENKTCL, NT), which is an extremely rare type of non-Hodgkin's lymphoma [5,6]. We report an exceptional case of primary testicular NK/T-cell lymphoma, nasal type, associated with high levels of EBV DNA load in plasma.

## Case presentation

**Figure 1:**
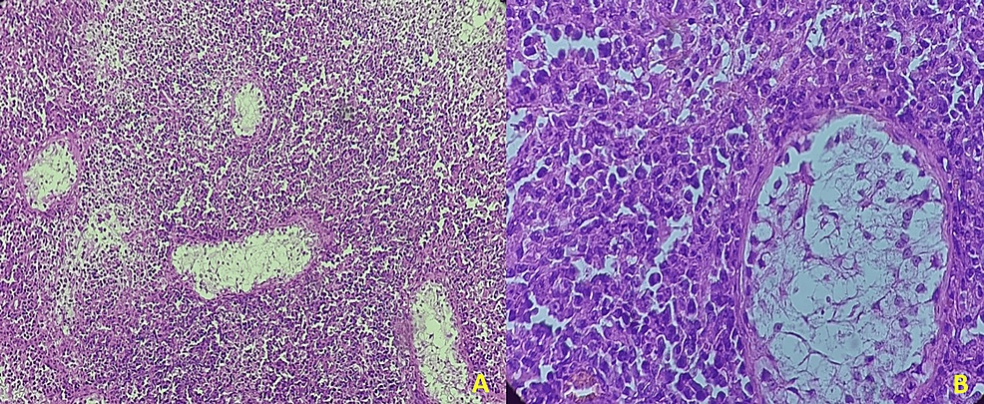
Histological aspects of extranodal T/NK lymphoma nasal type A (Low power): Lymphomatous proliferation with diffuse architecture dissociating the residual seminiferous tubules. B (High power): Medium to large tumor cells with folded nuclei and indistinct nucleoli with angiocentric pattern.

The patient was 45 years old. He had a history of treated syphilitic infection with no family history of neoplasia or hematologic malignancy. He had presented with painful testicular swelling associated with fever and a weight loss of 10 kg in 6 months. The abdominal examination revealed abdominal distension with hepatomegaly (16 cm) and splenomegaly. Lymph node examination revealed right and left supraclavicular adenopathies measuring 1 cm, firm, painless, and mobile in both planes and bilateral axillary adenopathies measuring 1 to 2 cm. An orchiectomy was performed, and a pathologic examination of the surgical specimen revealed a tumor cell proliferation. Histologically, the tumor cells were medium- to large-sized and arranged in diffuse sheets. They had ovoid nuclei with hyperchromatic irregular contours, no visible nucleoli, and numerous mitotic figures. This proliferation dissociated the residual seminiferous tubules (Figure 1). 

**Figure 2:**
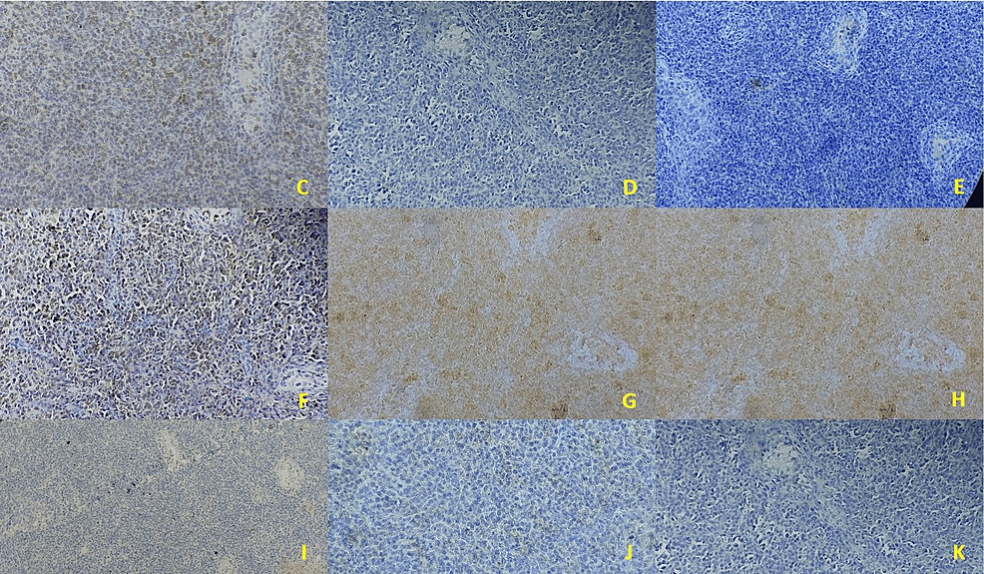
Immunohistochemical aspects of extranodal T/NK lymphoma nasal type Positive CD3 (C), positive Granzyme B (D), negative EBV (E), positive CD 56 (F), negative CD20 (G), negative TDT (H), negative BCL2 (I), negative CD30 (J), negative CD10(K).

An immunohistochemical study revealed positive anti-CD3 and anti-Granzyme B antibodies with negative anti-CD20, anti-CD5, anti-CD30, anti-BCL2, anti-CD10, anti-ALK, anti-Tdt, anti-EBV, anti-AE1/AE3, and anti-LMP1 antibodies in the tumor cells (Figure 2).

The Ki67 proliferation index was estimated at 80%. This profile was confirmed as testicular NK/T-cell lymphoma, nasal type. The patient was transferred to University Hospital's adult clinical hematology department in Rabat for treatment.

**Figure 3:**
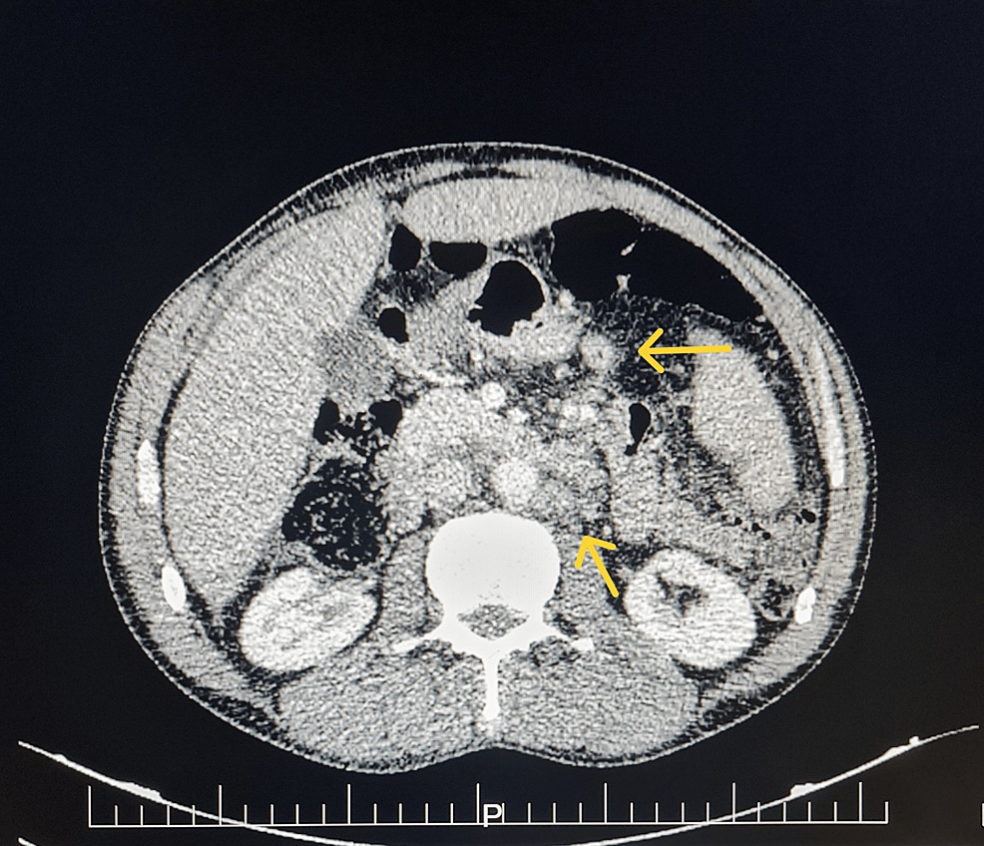
Axial CT images showing adenopathy below the diaphragm

Clinical examination revealed bilateral supraclavicular and axillary adenopathy with hepatosplenomegaly. A cervical-thoracic-abdominal-pelvic computed tomography scan revealed hepatosplenomegaly with adenopathy above and below the diaphragm (Figure 3).

Biological tests, including complete blood count, ionogram, renal function, and LDH, were normal. Liver function tests showed moderate cytolysis with AST=84 UI/L, ALT=75 UI/L, GGT=36 UI/L, and ALP=106 UI/L.

Quantification of EBV DNA by real-time PCR performed at the central virology laboratory at Ibn Sina Hospital in Rabat, using EBV ELITe MGB®/ELITe inGenius technology on a plasma sample, revealed a high level of EBV DNA load >1000000.00 IU/ml (> 6 log IU/ml) (Table 1).

**Table 1:** EBV DNA quantification limits on plasma using ELITe MGB®/ELITe inGenius technology

EBV DNA Quantification	UI/ml	Log UI/ml
Detection limit in plasma	124	2.09
Linearity	124 - 1000000	2.09 - 6

Quantification of CMV DNA using the same technique on the same plasma was negative. HBV serology revealed a profile of immunization by vaccination with negative HBs antigens, negative anti-HBc antibodies, and positive anti-HBs antibodies. HAV serology revealed an immunization profile. HCV and HIV serologies were negative.

All the clinical, radiological, biological, and pathological findings were in favor of primary testicular NK/T-cell lymphoma, nasal type, which was classified as stage IV according to the Ann Arbor classification.

The patient was treated with the SMILE chemotherapy protocol, which combined Dexamethasone; Methotrexate; Ifosfamide; L-asparaginase, and Etoposide. After four courses of SMILE chemotherapy, the patient showed complete remission on the PET scan, with EBV DNA levels decreasing to 2.74 log IU/ml and then becoming negative. An autograft was scheduled.

## Discussion

Epstein-Barr virus (EBV) is a ubiquitous, linear, double-stranded DNA herpes virus, transmitted mostly by the salivary route, with an affinity for epithelial cells of the oropharynx and B-lymphocytes of lymphoid tissue. After a primary infection, often asymptomatic in childhood, the virus persists in the body in a latent state, principally in the nuclei of memory B-lymphocytes in an episomal circular form [1,2].

In very rare situations, EBV can infect T lymphocytes and NK cells [5], and persist in the form of latency, generally type II [7]. In immunocompetent patients, this viral persistence can be responsible for malignant lymphoproliferations [1], including the nasal-type T/NK lymphoma diagnosed in our patient.

More common in Asia, Mexico, and South America [8], nasal-type LTNK is an "EBV-associated" non-Hodgkin's lymphoma, meaning that the viral genome is present in virtually all tumor cells [7]. Extra-nasal localization is exceptional. This is the case in our patients. Until 2018, only 20 cases of primitive testicular nasal-type NK/T cell lymphoma have been described [9,10].

The pathophysiological mechanism of these lymphomas is not very clear, but it may be explained by the genomic instability caused by EBV, which induces somatic mutations in oncogenic and tumor suppressor genes. The oncogenic proteins LMP1 and LMP2A also play a role in this process by promoting the proliferation of virus-infected cells or inhibiting their apoptosis [5]. Studies have shown that deficiency of RASGRP1 (RAS guanyl nucleotide-releasing protein), a nucleotide exchange factor specific to T lymphocytes, leads to defective proliferation of the T lymphocytes required for an effective immune response to EBV, which may explain an inherited susceptibility to EBV-induced lymphoproliferative disorders such as NK/T cell lymphoma [11,12].

Diagnosis of this highly aggressive tumor is based on immunohistochemistry. The NK phenotype is the most common. It is marked by the detection of cytoplasmic anti-CD3 antibodies -as was the case in our patient-, anti-CD2 and CD56 antibodies -not detected-, and the cytotoxic molecules TIA-1, perforin, and Granzyme B which was detected in our case [7].

The implication of EBV in this lymphoma is demonstrated by in situ hybridization, which shows the expression of EBV EBER transcripts by tumor cells [8]. Our patient did not have access to this test. Detection of EBV DNA load in plasma by real-time PCR, at the time of lymphoma diagnosis, shows high levels (Between 10^6^ and 10^9^ copies/mL), as is the case in our patient who presented a viral load> 6 log IU/ml. Consequently, the plasma EBV DNA load presents a useful biomarker in determining prognosis and monitoring the disease as well as assessing response to treatment [13,14]. Its presence is apparently associated with a poorer prognosis and has a negative impact on the survival of patients [9].

The SMILE chemotherapy protocol remains a useful therapeutic method that has proven its efficacy with a very high complete remission rate in patients with NK/T lymphoma [15]. This was the case with our patient. However, this does not exclude the possibility of relapse. In fact, the literature review shows that all patients with this lymphoma relapsed rapidly and died from complications of their disease, regardless of the modern treatment modalities administered, confirming the aggressiveness of the disease [9].

## Conclusions

Primary testicular NK/T-cell lymphoma, nasal type is uncommon. EBV is widely implicated in the pathophysiological process of this lymphoma type. Its quantification by real-time PCR remains one of the essential elements in the management of these lymphomas and in the therapeutic and prognostic follow-up of patients.
